# Effects of Traumatic Stress Induced in the Juvenile Period on the Expression of Gamma-Aminobutyric Acid Receptor Type A Subunits in Adult Rat Brain

**DOI:** 10.1155/2017/5715816

**Published:** 2017-03-02

**Authors:** Cui Yan Lu, De Xiang Liu, Hong Jiang, Fang Pan, Cyrus S. H. Ho, Roger C. M. Ho

**Affiliations:** ^1^Department of Medical Psychology, Shandong University School of Medicine, Jinan, Shandong 250012, China; ^2^Department of Psychological Medicine, National University of Singapore, Singapore 119228

## Abstract

Studies have found that early traumatic experience significantly increases the risk of posttraumatic stress disorder (PTSD). Gamma-aminobutyric acid (GABA) deficits were proposed to be implicated in development of PTSD, but the alterations of GABA receptor A (GABA_A_R) subunits induced by early traumatic stress have not been fully elucidated. Furthermore, previous studies suggested that exercise could be more effective than medications in reducing severity of anxiety and depression but the mechanism is unclear. This study used inescapable foot-shock to induce PTSD in juvenile rats and examined their emotional changes using open-field test and elevated plus maze, memory changes using Morris water maze, and the expression of GABA_A_R subunits (*γ*2, *α*2, and *α*5) in subregions of the brain in the adulthood using western blotting and immunohistochemistry. We aimed to observe the role of GABA_A_R subunits changes induced by juvenile trauma in the pathogenesis of subsequent PTSD in adulthood. In addition, we investigated the protective effects of exercise for 6 weeks and benzodiazepine (clonazepam) for 2 weeks. This study found that juvenile traumatic stress induced chronic anxiety and spatial memory loss and reduced expression of GABA_A_R subunits in the adult rat brains. Furthermore, exercise led to significant improvement as compared to short-term BZ treatment.

## 1. Introduction

Posttraumatic stress disorder (PTSD) is an anxiety disorder that occurs following exposure to severe trauma. In humans, early traumatic experience and adversity significantly increase vulnerability to psychiatric disorders including depression and PTSD in adulthood [1, 2]. Studies in animals showed that the experience of early traumatic events including maternal separation, postnatal neglect, and abuse could alter behavioral reaction, neuroendocrine responsiveness, and brain morphology, thereby increasing the risk of PTSD [3]. Studies found that GABA, an inhibitory neurotransmitter in mammalian brain, was involved in traumatic stress responses. Nevertheless, few studies investigated the influence of adverse experience encountered in the early life periods on the GABAergic system and PTSD in adulthood [4].

The GABAergic system plays a role in the pathogenesis of anxiety disorders [5, 6]. Recent studies found that deficits in GABAergic transmission in the brain might be involved in the pathophysiology of anxiety disorders including PTSD [7, 8]. GABA exerts its major function through the GABA type A receptors (GABA_A_Rs), which inhibits hyperarousal state and anxiety. Clonazepam, a commonly used benzodiazepine (BZ), demonstrates positive effects in improving anxiety and sleep disturbances in PTSD by binding to the BZ site of GABA_A_Rs and strengthens inhibitory neurotransmission [9]. The GABA_A_Rs are composed of two *α* subunits, two *β* subunits, and one *γ* subunit. The *γ*2 subunit is most commonly found in the brain and facilitates the binding of BZs to the GABA_A_R [10]. The *α* subunit is associated with various pharmacological properties of BZ [11]. The *α*1 subunit of GABA_A_R induces the sedative, amnestic, and anticonvulsant effects [12, 13]. The *α*2 and *α*3 subunits are involved in the anxiolytic action [14]. The *α*5 subunit is involved in formation of hippocampus-dependent associative memory and muscle relaxant effects [12, 15]. The *α*2 subunit is highly expressed in the corticolimbic regions, whereas *α*5 subunit is densely located in the hippocampus. Recently, neuroimaging studies identified abnormality in the corticolimbic circuit including the prefrontal cortex (PFC), amygdala, and hippocampus in patients suffering from PTSD [16, 17]. Deficits in GABAergic neurotransmission in the corticolimbic regions might be one of the predisposing factors for PTSD. Nevertheless, the alternations of GABA_A_R subunits expression induced by early traumatic experience and its role in vulnerability to PTSD in adulthood are not clearly elucidated.

Previous research demonstrated positive effects of exercise on physical symptoms (e.g., dyspnea pain, fatigue) [18] and incidence of psychiatric disorders in adolescents and young adults [19]. Vujanovic et al. (2013) found that the levels of weekly exercise demonstrated an inverse relationship with anxiety-like or PTSD symptoms [20]. Exercise was found to have similar efficacy to antidepressants in relieving anxiety and depressive symptoms [21]. The underlying neurobiological mechanisms behind the effects of exercise in reducing anxiety-like or PTSD symptoms remain unknown.

In this study, inescapable foot shook was used to induce PTSD in juvenile rats. We examined changes in animal behaviors and expressions of the GABA_A_R *α*2, *α*5, and *γ*2 subunits in three corticolimbic regions (i.e., the prefrontal cortex (PFC), amygdala, and hippocampus) of adult rat brains. The intervention of PTSD was composed of 6-week exercise and 2-week clonazepam treatment. The first hypothesis of the study was that early traumatic stress would induce changes of the GABA_A_R subunits (*α*2, *α*5, and *γ*2) expression in the corticolimbic regions, resulting in deficits in GABAergic neurotransmission which increase vulnerability to PTSD in adulthood. The second hypothesis was that long-term exercise and clonazepam treatment would improve PTSD symptoms and increase expression of GABA_A_R subunits in adult rat brains.

## 2. Materials and Methods

### 2.1. Experimental Animals and Grouping

A total of 48 male Wistar rats (postnatal day: 21 day) were received from the experimental animal center of Shandong University, China. The rats were housed in cages (six per cage) in a temperature- and humidity-controlled environment, under a 12-hour dark-light cycle (lights on 6 AM–6 PM), and food and water ad libitum were used. Animals were acclimated to these housing conditions for 1 week before any experimental manipulation. All procedures in this study were approved by the Ethics Committee of the School of Medicine, Shandong University, which complied with the National Institute of Health Guide for the Care and Use of Laboratory Animals (NIH publication number 85-23, revised 1985).

The rats were randomly divided into 4 groups (*n* = 12 in each group, six per cage): the control group (C), traumatic stress group (T), traumatic stress and exercise group (T + E), and traumatic stress and drug (BZ, clonazepam) treatment group (T + D). Traumatic stress groups received traumatic stress procedure for 7 consecutive days at postnatal days 28 to 34 (i.e., the juvenile period). The T + E group underwent treadmill run for 6 weeks and T + D group received drug treatment for 2 weeks on the following day after the traumatic stress procedure was completed.

### 2.2. Animal Model of PTSD

Rats were exposed to repeated inescapable electric foot-shock for 7 consecutive days according to methods used in previous study [22]. The rats were put in a closed, dark electric box with electrified bottom and received 0.2 mA electric foot-shock, which persisted 6 seconds, was repeated 10 times with a 30 sec interval between each trial, and was repeated for three trials per day.

### 2.3. Treadmill Exercise Procedure

The regular, long-term exercise protocol in our study was modified from a previous study [23]. Rats were trained to run on a motorized treadmill once a day for 6 weeks. The treadmill has six channels of plastic boards and each channel is 80 cm × 20 cm × 10 cm. The rats ran at a speed of 9 m/min, 10 min/day, in the 1st week. In the following 5 weeks, the speed was increased to 12 and 15 m/min during the 3rd and 5th week, respectively. The duration was gradually increased to 60 min during the 6th week. The animals in nonexercise groups were left on the treadmill without running for the same time.

### 2.4. Administration of Clonazepam

Clonazepam was administrated orally with 0.5 mg/kg/day (in distilled water) based on the dosage used in previous study [24] for 2 weeks after the completion of electric foot-shock procedure. Clonazepam was obtained from Qilu Hospital, China. In order to expose all animals to the same manipulation procedures, animals in other groups were exposed to oral gavages of distilled water for 2 weeks.

### 2.5. Behavioral Testing

Behavioral tests were conducted on the following day after the exercise procedure was completed. The order of behavioral tests was as follows: (1) open-field test, (2) elevated plus maze test, and (3) Morris water maze test.

#### 2.5.1. Open-Field Test (OFT)

Exploratory behavior and emotional reaction were observed in the OFT. The OFT was conducted based on our laboratory procedure which was published in a previous study [25]. The apparatus (90 cm × 90 cm × 45 cm) was divided into 25 equal squares. Rats in each group were put in the center of the open-field and observed for 5 minutes. The duration of time spent in the center squares (squares except peripheral squares along the side), line crossings (frequency with which the rat crossed one of the grid lines with all 4 paws), frequency of grooming (the number of times when the rat spent licking or scratching itself while stationary), and frequency of rearing (frequency with which the rat stood on their hind legs on the ground) were recorded for 5 minutes by the SMART video tracking system (SMART v3.0, Panlab, Spain). The low frequencies of the above behaviors indicated low levels of locomotion and exploration but high level of anxiety.

#### 2.5.2. Elevated Plus Maze (EPM)

Anxiety-like behaviors were measured in the EPM. The maze was elevated 50 cm above the floor and consisted of a central part (5 cm × 5 cm), two opposing open arms (30 cm × 5 cm), and two opposing closed arms (30 cm × 5 cm) with a 15 cm height. Each animal was placed in the central part of the maze facing an open arm and allowed free exploration for 5 minutes under ambient room light. The maze was cleaned with 75% ethanol and dried between usage. All processes were videotaped, and the number of entries to open and closed arms and the time spent in each arm were measured by the SMART video tracking system (SMART v3.0, Panlab, Spain). The lower percent of time and entries in the open arm indicates more anxiety-like behavior in rats.

#### 2.5.3. Morris Water Maze (MWM)

Spatial learning ability was evaluated in the MWM. The water maze was a circular pool (140 cm in diameter, and equipped with a platform 1-2 cm below the water surface) and filled with water at 22 ± 2°C. Visible cues in the room were provided for orientation. Rats were placed in water for 60 seconds for habituation before the start of the test. The test consisted of the acquisition phase during the first five days and the probe trial on the sixth day. In the acquisition phase, the rat had four trials in each day for 5 consecutive days to find the platform. In each trial, the rat was put in the water facing the wall of the pool in one of the four quadrants. The rat was allowed to look for the platform for 60 sec. If it managed to find the platform, it would remain on the platform for 20 seconds. If it could not find the platform within 60 seconds, it would be guided by the experimenter to find the platform and then it remained on the platform for 20 seconds. Once each training session had finished, the rat would be dried by a towel and sent back to its cage. In the probe trial to test memory, the platform was removed from the pool. The rats were placed in water for 60 sec for one trial. The number of crossings of the former platform location (target area) and the time spent in the target quadrant were measured by the SMART video tracking system (SMART v3.0, Panlab, Spain).

### 2.6. Western Blotting (WB)

The brains of rats were removed after the rats were decapitated. Tissues of the PFC, hippocampus, and amygdala were separated bilaterally, weighed, and frozen immediately in liquid nitrogen and then kept at −80°C in refrigerator for protein isolation for subsequent examining of the GABA_A_R subunits expression (*α*5 was examined only in the hippocampus as its preponderant expression in it).

Tissue samples were homogenized in lysis buffer containing 1% protease inhibitor phenylmethanesulfonyl fluoride (PMSF). The homogenate was centrifuged at 12000*g* for 10 min at 4°C, and then the supernatant was obtained. The protein concentration was determined using a microbicinchoninic acid (BCA) Protein Assay Kit (Beyotime Institute of Biotechnology). Samples containing equal amounts of proteins were mixed with a 6x Laemmli loading buffer (50 mM Tris-HCl, PH 6.8; 2% sodium dodecyl sulfate (SDS); 10% glycerol; 0.02 mg/mL bromophenol blue; and 0.1 M dithiothreitol, pH 6.8). After the denaturing and reducing conditions, protein samples (25 *μ*g) were separated with 5–12% sodium dodecyl sulfate-polyacrylamide (SDS-PAGE) gel and transferred to polyvinylidene difluoride (PVDF) membranes (Bio-Rad, CA, USA). Then the membrane was blocked with 5% bovine serum albumin (BSA) in TBST solution containing 0.05% Tween 20 for 1 hour and incubated with primary polyclonal antibodies against GABA_A_R *γ*2 (1 : 1000, 45 kDa), *α*2 (1 : 1000, 51 kDa), and *α*5 (1 : 1000, 52 kDa) subunits (anti-rabbit, NOVUS, USA) or GAPDH (anti-mouse, 1 : 8000, Biogot Technology, Co, Ltd) overnight at 4°C. On the second day, the membrane was washed five times with Tris-buffered saline and Tween 20 (TBST) and incubated with appropriate horseradish peroxidase- (HRP-) conjugated secondary antibody (1 : 10000) for 1 hour at room temperature. After washing four times with TBST, the bands were visualized in ECL solution (Millipore Corp., Billerica, Massachusetts, USA) for 5 min and exposed onto films. The band density value of individual proteins was normalized with reference to that of the GAPDH of the same sample. Signal intensities were quantified using the Image J 14.0 software.

### 2.7. Immunohistochemistry (IHC)

Six rats from each group were anesthetized using sodium pentobarbital (40 mg/kg). The hearts were exposed and perfused with 50 mM phosphate-buffered saline (PBS), then with a freshly prepared solution containing 4% paraformaldehyde in 100 mM phosphate buffer (PB, pH 7.4) for fixation. After perfusion, whole brains were removed rapidly and immersed in 4% paraformaldehyde (PFA) for 24 hours at 4°C. Then, the brain samples were dehydrated and embedded in paraffin wax with a coronal orientation. The PFC (from bregma 4.2 mm to 4.7 mm) and hippocampus (from bregma −3.3 mm to −3.1 mm) were cut into slices with 5 *μ*m thickness.

The sections were dewaxed and incubated in 3% H_2_O_2_ for 10 min at room temperature, antigens were repaired in 0.01 M citrate buffer (pH 6.0) with a microwave, blocked for 20 minutes with goat serum, and incubated with rabbit anti-GABA_A_R subunits antibody at 4°C overnight as follows: anti-*γ*2, 1 : 500, anti-*α*2, 1 : 500, and anti-*α*5, 1 : 600. The following day, biotinylated secondary antibody was added to the sections and incubated for 30 min at 37°C, followed by Streptavidin-Biotin Complex (SABC) for 30 min at 37°C, and then stained with diaminobenzidine (DAB) under the microscope. The pictures were photographed with a digital camera (Zeiss Axiocam MRc5).

### 2.8. Statistical Analysis

All data were analyzed using the one-way ANOVA followed by Bonferroni's post hoc test by the statistical software SPSS 16.0. The results were expressed as the mean ± standard error of the mean (SEM). Significance was accepted to be present at *p* < 0.05.

## 3. Results

### 3.1. Anxiety-Like Behaviors and Spatial Memory Performance

#### 3.1.1. Results of the OFT

**Figure 1:**
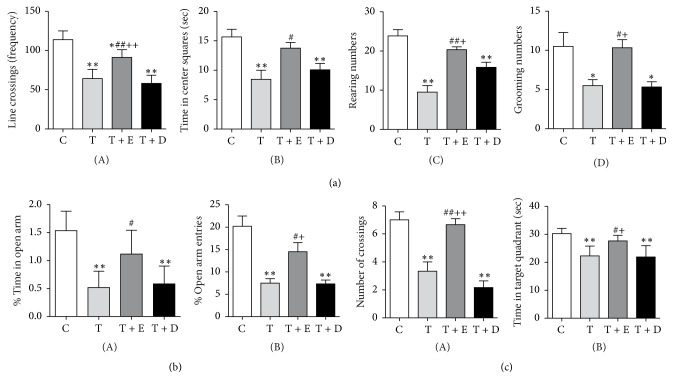
Anxiety-like behaviors and spatial memory performance. (a) Behavioral changes in the open-field test (*n* = 12, per group): (A) frequency of line crossing, (B) time spent in central squares, (C) rearing times, and (D) grooming numbers. (b) Behavioral results in the elevated plus maze (*n* = 12, per group): (A) percentage of time in open arm and (B) percentage of open arm entries. (c) Spatial memory performance in the Morris water maze (*n* = 12, per group): (A) number of crossings in the target area and (B) time spent in the target quadrant. Values were expressed as mean ± SEM. ^*∗*^*p* < 0.05 and ^*∗∗*^*p* < 0.01 versus C group; ^#^*p* < 0.05 and ^##^*p* < 0.01 versus T group; ^+^*p* < 0.05 and ^++^*p* < 0.01 versus T + D group.

Figure 1(a)(A) shows the frequency of line crossing in each group and there were significant differences among groups [*F*(3, 44) = 33.600, *p* < 0.001]. Compared to rats in the C and T + E groups, rats in the T (*p* < 0.001, *p* < 0.05) and T + D (*p* < 0.001, *p* < 0.001) groups had less frequent line crossings. Rats in T + E group had less frequent line crossings than rats in the C group (*p* < 0.05). Figure 1(a)(B) shows the time rats in each group spent in the center squares and there were significant differences among groups [*F*(3, 44) = 7.034, *p* < 0.05]. The T group spent significantly less time in the center squares than the C and T + E (*p* < 0.001, *p* < 0.05) groups. Rats in the T + D group spent less time in the center squares than the C group (*p* < 0.001). Figure 1(a)(C) shows the rearing numbers of each group and there were significant differences among groups [*F*(3, 44) = 20.093, *p* < 0.001]. Rats in the T and T + D groups displayed lower rearing numbers than rats in the C group (*p* < 0.001, *p* < 0.001), whereas rats in the T + E and T + D groups demonstrated significantly higher rearing numbers than rats in the T group (*p* < 0.001, *p* < 0.05). Figure 1(a)(D) shows grooming numbers of each group and there were significant differences among groups [*F*(3, 44) = 6.373, *p* < 0.05]. The C and T + E groups had higher grooming numbers than the T (*p* < 0.05, *p* < 0.05) and T + D groups (*p* < 0.05, *p* < 0.05).

#### 3.1.2. Results of the EPM

Figure 1(b)(A) displays the percent time spent in the open arm of each group [*F*(3, 44) = 13.736, *p* < 0.001]. Rats in the T and T + D groups demonstrated significantly lower percent time in the open arm than the C group (*p* < 0.001, *p* < 0.001). Rats in the T group showed significantly lower percent time than the T + E group (*p* < 0.05). Figure 1(b)(B) shows the percent entries to the open arm of each group and there were significant differences among groups [*F*(3, 44) = 11.221, *p* < 0.001]. The T rats displayed lower percent entries than the C and T + E groups (*p* < 0.001, *p* < 0.001). Rats in the T + D group had lower percent entries as compared to the C and T + E groups (*p* < 0.001, *p* < 0.05).

#### 3.1.3. Results of the MWM

Figure 1(c) (A) shows the number of crossings in the target area in each group and there were significant differences among groups [*F*(3, 44) = 19.617, *p* < 0.001]. Rats in the T and T + D groups demonstrated significantly lower number of crossings than rats in the C (*p* < 0.001, *p* < 0.001) and T + E (*p* < 0.001, *p* < 0.001) groups. Figure 1(c)(B) shows the time spent in the target quadrant of each group and there were significant differences among groups [*F*(3, 44) = 11.250, *p* < 0.001]. Compared to the C and T + E groups, rats in the T (*p* < 0.001, *p* < 0.05) and T + D (*p* < 0.001, *p* < 0.05) groups spent less time in the target quadrant.

#### 3.1.4. GABA_A_R Subunits Expressions of Western Blotting

**Figure 2:**
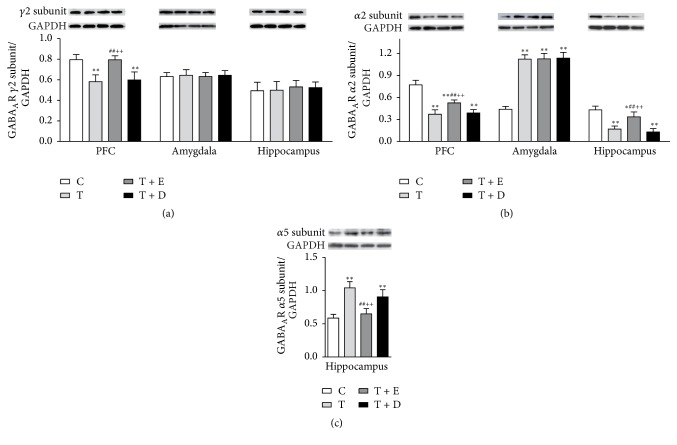
GABA_A_R subunits expressions in subregions of brain in western blotting. (a) The expression of *γ*2 subunit in the PFC, amygdala, and hippocampus. (b) The expression of *α*2 subunit in the PFC, amygdala, and hippocampus. (c) The expression of *α*5 subunit in the hippocampus. Values were expressed as mean ± SEM (*n* = 6, per group). ^*∗*^*p* < 0.05 and ^*∗∗*^*p* < 0.01 versus C group; ^##^*p* < 0.01 versus T group; ^++^*p* < 0.01 versus T + D group.

Figure 2(a) shows the *γ*2 subunit expressions of rats in each group in the PFC, amygdala, and hippocampus. There were significant differences of *γ*2 subunit expression in the PFC among four groups [*F*(3, 20) = 22.895, *p* < 0.001]. Rats in the T and T + D groups showed lower expression than rats in the C (*p* < 0.001, *p* < 0.001) and T + E (*p* < 0.001, *p* < 0.001) groups. No significant differences in expressions of *γ*2 subunit were found between four groups in the amygdala [*F*(3, 20) = 0.399, *p* > 0.05] and hippocampus [*F*(3, 20) = 0.166, *p* > 0.05].  Figure 2(b) shows the *α*2 subunit expression in each group and there were significant differences among groups in the PFC, amygdala, and hippocampus [*F*(3, 20) = 71.894, 166.099, 44.878, *p* < 0.001]. In the PFC, the expression of *α*2 subunit in rats of the T and T + D groups was significantly lower than rats of the C (*p* < 0.001, *p* < 0.001) and T + E (*p* < 0.001, *p* < 0.001) groups. Meanwhile, rats in the T + E group had lower expression of *α*2 subunit than rats in the C group (*p* < 0.001). In the amygdala, rats in the T, T + E, and T + D groups had significantly higher expression of *α*2 subunit expression as compared with the C group separately (*p* < 0.001, *p* < 0.001, and *p* < 0.001). In the hippocampus, the expression of *α*2 subunit of the T and T + D groups was significantly lower than the C (*p* < 0.001, *p* < 0.001) and T + E groups (*p* < 0.001, *p* < 0.001). The expression of *α*2 subunit of the T + E group was lower than rats in the C group (*p* < 0.05).  Figure 2(c) shows expression of *α*5 subunit expression in the hippocampus for each group and there were significant differences among groups [*F*(3, 20) = 36.780, *p* < 0.001]. Rats from the T and T + D groups had significantly higher expression of the *α*5 subunit expression than the C (*p* < 0.001, *p* < 0.001) and T + E (*p* < 0.001, *p* < 0.001) groups.

#### 3.1.5. IHC Results

**Figure 3:**
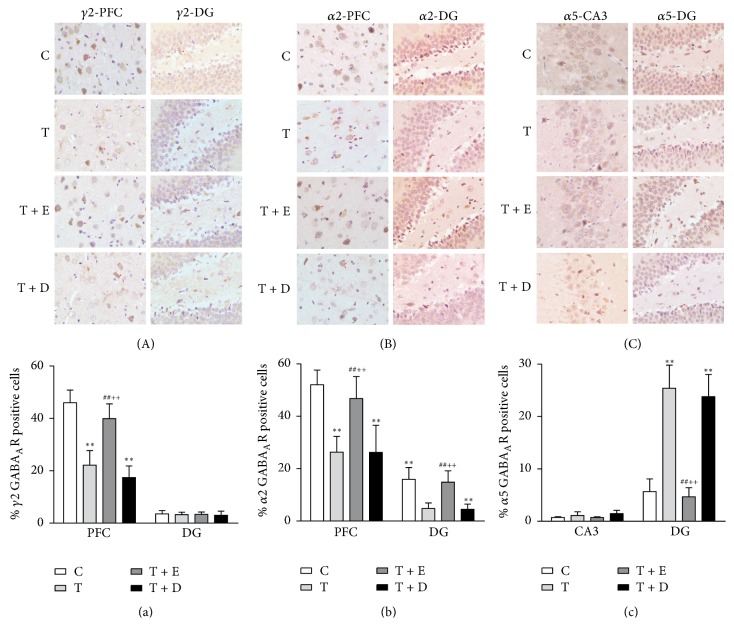
Representative expression of the GABA_A_R subunits in the PFC, CA3, and dentate gyrus (DG) of the hippocampus (×400). The neurons with positive *γ*2 subunit expression and the results of positive cell counting in the PFC and DG were shown in (A) and (a). The neurons with positive *α*2 subunit expression and the results of positive cell counting in the PFC and DG were shown in (B) and (b). The neurons with positive *α*5 subunit expression and the results of positive cell counting in the DG and CA3 subregions of the hippocampus were shown in (C) and (c). Values were expressed as mean ± SEM (*n* = 6, per group). ^*∗∗*^*p* < 0.01 versus C group; ^##^*p* < 0.01 versus T group; ^++^*p* < 0.01 versus T + D group.

The immunohistochemical picture (see Figures 3(A)–3(C)) shows expression of GABA_A_R subunits visually in the PFC, CA3, and the dentate gyrus (DG) of hippocampus under the high-power microscope (×400). The positive cells displayed brownish yellow granules on the membrane. Figures 3(a)–3(c) showed the results of GABA_A_R subunits positive cell counting in groups. The results showed that there were significant differences in the expression of *γ*2 [*F*(3,20) = 43.083, *p* < 0.001] and *α*2 subunit [*F*(3,20) = 17.995, *p* < 0.001] in the PFC among the 4 groups. Fewer neurons with positive *γ*2 and *α*2 subunit expression were observed and measured in the T and T + D groups as compared with the C (*p* < 0.001, *p* < 0.001) and T + E (*p* < 0.001, *p* < 0.001) groups. There was no significant difference [*F*(3, 20) = 0.939, *p* > 0.05] in the percentage of *γ*2 subunit positive cells in the DG of hippocampus in the four groups. There were significant differences in the expression of *α*2 in the DG region of hippocampus among the 4 groups [*F*(3, 20) = 19.764, *p* < 0.001]. The percentage of *α*2 subunit positive cells of the T and T + D groups was significantly lower than that of the C (*p* < 0.001; *p* < 0.001) and T + E groups (*p* < 0.001, *p* < 0.001). In contrast, the expression and results of positive cells of *α*5 subunit were significantly higher in the DG of hippocampus [*F*(3, 20) = 65.972, *p* < 0.001] in the T and T + D groups as compared with the C (*p* < 0.001, *p* < 0.001) and T + E groups (*p* < 0.001, *p* < 0.001). No significant difference of *α*5 subunit expression was observed in the CA3 subregion of hippocampus among the 4 groups [*F*(3, 20) = 3.453, *p* > 0.05].

## 4. Discussion

This animal study demonstrated that traumatic stress in the juvenile period induced persistent changes in emotional behavior, spatial memory, and the expression of GABA_A_R subunits in the corticolimbic regions during adulthood, which provide further understanding behind the vulnerability to PTSD for humans.

Previous studies showed that childhood adversity induced increased anxiety [26] and depression-like behavior [27, 28] and damaged spatial learning and memory [26, 29]. Our study showed that exposure to traumatic stress in the juvenile period induced anxiety-like behavior as evidenced by significantly lower line crossings, shorter time spent in central squares, lower number of grooming and rearing instances during the OFT, and significantly lower percent of entries to the open arm and percent of time spent in the open arm during the EPM in rats exposed to trauma as compared to controls. Rats exposed to trauma also demonstrated impaired learning and memory, and this was represented by significantly lower number of platform crossings and shorter time spent on the target quadrant in the MWM as compared to controls. These results supported that our animal model was valid to demonstrate that traumatic stress in early life led to behavioral dysfunction during adulthood. Our study also demonstrated that exercise could reduce the anxiety-like behaviors during adulthood in rats exposed to early trauma during juvenile period, but administration of BZ could not reduce most of the anxiety-like behaviors. In this study, the duration of clonazepam treatment was based on previous studies. Clinical studies showed that the most suitable duration of clonazepam treatment for anxiety disorders is 2–4 weeks to avoid dependence [30]. In animals, the levels of locomotor activity which signify anxiety were reduced by 2–14 days of benzodiazepines treatment [31, 32]. For the duration of exercise, previous study suggested that progressively increasing moderate exercise could reduce stress reaction [33] and depression-like behavior of rats exposed to early life maternal deprivation [23]. Another study found that long-term exercise (6 weeks) could reverse PTSD-like behaviors in rats [34]. Our results support the previous findings that long-term exercise could reduce the anxiety-like behavior and posttraumatic stress symptoms in animals exposed to traumatic stress during juvenile period [34].

Studies demonstrated that patients with PTSD have abnormalities in the corticolimbic circuit including the PFC, amygdala, and hippocampus. These neuroanatomical structures are implicated in the supposed fear learning abnormalities and sensitization reported in PTSD [35]. Some studies reported that there was reduced BZ-GABA_A_R binding in veterans with PTSD [7] and decreased BZ receptors in the PFC in rats suffering from early trauma [36]. However, the effect of early exposure to traumatic stress during juvenile period on the GABA_A_R and its implication in pathogenesis of PTSD in adults remains unknown. Our results shed light on the pathogenesis and found that the expression of GABA_A_R *γ*2 subunit was decreased in the PFC in rats exposed to early trauma as compared to controls. It is well known that the *γ*2 subunit is required for binding of BZ. Reduced expressions of *γ*2 subunit in stressed animals result in decreased binding of BZs and increased anxiety level. The *γ*2 subunit mediates fast synaptic inhibition and leads to functional deficits in GABA_A_Rs and anxiety symptoms [37]. Our results indicated that the decreased expression of *γ*2 subunit may lead to deficits in GABA transmission and synaptic plasticity in the PFC [38]. Our results support the previous view that a reduced number of GABA_A_R binding sites play a role for GABAergic deficits in anxiety disorders [8]. Furthermore, the PFC controls stress response by inhibitory GABAergic projections to the amygdala and plays an important role in emotional regulation [39]. Therefore, the GABA_A_R deficits in the PFC would lead to its weaker upstream control over downstream neuroanatomical regions in the corticolimbic circuits and results in anxiety-like behavior.

Previous studies identified the dominant localization of *α*2 subunit in the synapses of the PFC and hippocampus which was thought to exert influences on the output activities of synapses [40]. Our data showed that traumatic stress during the juvenile period decreased the expression of *α*2 subunit levels in the PFC and hippocampus and increased the expression of *α*2 subunit levels in the amygdala. This finding requires further interpretation. Insufficient top-down control from the PFC to the amygdala results in enhanced activity of amygdala and emotional dysregulation [41]. One study found that stress increased *α*2 subunit in the basolateral amygdala of rats [42], which leads to anxiety-like and fearful responses. Hence, the reduced expression of *α*2 subunit in the PFC is associated with failure in elimination of fear-related memories [43, 44] and attenuated control over fear responses in PTSD [45]. Our results suggested that early exposure to traumatic stress induced regional alternations of *α*2 subunit expression in the rat brains which exert different modulation over the hypothalamus by direct or indirect GABAergic pathway [4].

GABA_A_R *α*5 subunit is predominantly expressed in the hippocampus [46]. Evidence shows that *α*5 subunit is involved in hippocampus-independent spatial memory [47]. Reduced *α*5 subunit expression is linked to facilitated cognition in hippocampal-dependent tasks [48, 49] and reduction of *α*5-GABA_A_R function is regarded as a cognition improving strategy for dementia and cognitive impairment [50]. Our study found that the expression of *α*5 subunit was significantly increased in rats exposure to early trauma with poor spatial memory performance in the MWM test. Our results suggest that the increased expression of GABA_A_R *α*5 subunits may be the underlying pathology behind poor memory observed in patients suffering from PTSD.

Clonazepam enhances the function of GABA neurotransmission by the binding to specific BZ recognition sites of GABA_A_R. Loewenstein (1991) reported that clonazepam alleviated severity of insomnia, nightmares, and panic attacks in PTSD patients [51]. Nevertheless, studies showed that no distinguishing effects were found between BZs and placebo in treating PTSD in the short-term and consuming BZs cannot prevent development of PTSD in the long term [52–54]. Recently, researchers proposed that early administration of BZ could inhibit normal response in the HPA axis and facilitate the development of PTSD [55]. Currently, BZs are usually administered for a time-limited period to avoid potential dependence and addiction. Some studies were conducted to observe long-term effects of BZ administration on expression of the GABA_A_R subunits [56]. Our results showed that rats exposed to early traumatic stress and receiving clonazepam treatment did not display amelioration of anxiety-like behavior, memory loss, and alteration of expression of the GABA_A_R subunits compared with rats exposed to early trauma but receiving no treatment. Our findings suggest that BZ treatment alone may not fully relieve PTSD symptoms and reverse the underlying pathophysiology. Further studies are required to evaluate the effect of high dose clonazepam on GABA_A_R subunits in the treatment of PTSD.

It is well known that exercise could improve anxiety, depression, and cognition in humans [57]. Dishman et al. [58] found that exercise reduced anxiety by decreasing the binding affinity of GABA_A_R in the corpus striatum in rats. Our study was the first to report that treadmill exercise could increase the expression of *γ*2 subunits in the PFC and *α*2 subunit expressions in the PFC and hippocampus and decreased the expression of *α*5 subunit in the hippocampus after early exposure to traumatic stress. Our results provide new neurobiological evidences to support nonpharmacological treatment for PTSD.

This study has several limitations. First, we could not study the long-term effects of BZ on the expression of GABA_A_ receptor subunits. Second, a single dose of clonazepam was administered based on the body weight. Higher doses of clonazepam might reduce expression of *α*2 subunits in the amygdala after exposure to early life stress. BZ treatment may have the capacity to alter the pattern of expression of certain subunits and further research is required. Third, several reports suggest the role of the *α*5 subunit in mediating the tonic current in the hippocampal neurons [59, 60]. In this study, we did not perform Hydrophobic Interaction Chromatography (HIC) experiments. Further study is required to perform HIC experiments in pyramidal neurons of the CA1 field.

## 5. Conclusions

Our results showed that early traumatic stress induced persistent effect on negative emotion and spatial memory and region-specific alterations of GABA_A_R *γ*2, *α*2, and *α*5 subunit expression of brain in adulthood which may contribute to dysregulated responsivity to stress and increased susceptibility to PTSD. Long-term exercise could alleviate the influence from early trauma on behavior and the expression of GABA_A_R subunits, but short-term BZ treatment did not demonstrate such effect.

## Abbreviations

PTSD: Posttraumatic stress disorder
HPA: Hypothalamic pituitary adrenal
PFC: Prefrontal cortex
GABA: Gamma-aminobutyric acid
GABA_A_R: GABA receptor A
BZs: Benzodiazepines
OFT: Open-field test
EPM: Elevated plus maze
MWM: Morris water maze.
